# Editorial: New insights into the role of the vagus nerve in health and disease: Basic and clinical studies

**DOI:** 10.3389/fnins.2022.979695

**Published:** 2022-09-28

**Authors:** Vitor E. Valenti

**Affiliations:** Autonomic Nervous System Center (CESNA), São Paulo State University (UNESP), Marília, SP, Brazil

**Keywords:** autonomic nervous, vagus, vagus afferent nerves, parasympathethic tone, parasympathetic

The vagus nerve plays an important role in the homeostatic control of several organ systems in health and disease (Karemaker, 2022). This Research Topic presents some of the latest work that explores different aspects of vagal control.

Kuchler et al. analyzed the role of the spleen in vagal activity and metabolism. The mentioned experimental study assessed the impact of vagotomy associated with splenectomy on white adipose tissue in obese rats. It is worth noting that obesity was induced *via* hypothalamic lesions, leading to lifelong obesity. The main finding of the study suggested that vagal-splenic mechanisms control the metabolism in health conditions.

Kobrzycka et al. conducted research on the hypothalamus. The researchers performed vagotomy in rats to understand the role of the vagus nerve on neurochemical alterations in the hypothalamus. The results indicated that, although mRNA expression was not changed, vagotomy affected the hypothalamic amino acids in the long term.

The study conducted by Yu et al. evaluated the interaction between transcutaneous auricular vagal nerve stimulation and cerebral hemodynamics in consciousness restoration. This open-label pilot clinical trial performed functional magnetic resonance imaging to analyze cerebral hemodynamics. The results indicated that this specific vagal stimulation technique activated the interoceptive and limbic systems, suggesting disorders of consciousness attenuation.

Due to the abovementioned limitation, Osińska et al. conducted a longitudinal case study to evaluate patients with disorders of consciousness submitted to vagal nerve stimulation. Electrophysiological measures included electroencephalography and heart rate variability. The authors reported that the 6-month intervention was able to improve the behavioral indices of consciousness and reinforced that the alpha wave electroencephalography power gradually increased.

Moreover, auricular vagal stimulation was analyzed during the COVID-19 pandemic. The abovementioned mentioned multicentric, randomized, controlled, double-blind study investigated the influence of auricular neuromodulation through auricular acupuncture using semipermanent needles on clinical outcomes in patients with COVID-19 (Rangon et al.). The outcome measures included the seven-category ordinal scale, time until clinical improvement, and transfer to the intensive care unit. Unfortunately, the authors did not observe a significant beneficial effect of this intervention on the outcomes of the COVID-19 patients.

Our Research Topic also explored psychiatric comorbidities and their association with autonomic reactivity. Ruschil et al. focused on heart rate variability, a non-invasive method that estimates vagal control of heart rhythm (Kloter et al., 2018). The authors examined neurological patients with medically unexplained sensory symptoms and analyzed the regulation of their HRV in different conditions, i.e., placebo application, cold-face test, and pain stimuli. The data pointed to altered vagal function in this specific condition.

The association between the vagus nerve, autonomic activity, and fetal development was investigated by Cerritelli et al. The authors conducted a simple review and highlighted the importance of non-invasive assessment of maternal and fetal autonomic function through heart rate variability. The authors reinforced that monitoring health status throughout the pregnancy, from its earliest stages, is necessary to identify risks and outcome projections.

In this line, the association between the vagus nerve and bowel inflammation was investigated by Caravaca et al. The bowel disease model was based on an experimental indomethacin-induced acute intestinal inflammation in male Sprague Dawley rats. Vagal activation was performed *via* the left cervical vagus nerve, and the splenic nerve was also stimulated. Reduction of the TNF levels following vagal nerve stimulation evidenced that this intervention provided a benefic impact on small bowel inflammation.

Cardiac arrest, a very interesting issue, was also explored in our Research Topic. Kim et al. observed the effects of vagal nerve stimulation on cerebral mitochondrial dysfunction in an asphyxial cardiac arrest rat model. The respiration measurement in mitochondria was necessary to better understand this mechanism. The study evidenced that the stimulation of the cervical vagus nerve improved cerebral injury in male Sprague–Dawley rats.

Finally, a sophisticated investigation analyzed the spatial working memory in young adults who submitted to vagal stimulation (Sun et al.). The authors performed transcutaneous auricular vagus nerve stimulation to activate the vagus nerve and working memory tasks to better understand memory status. Among the main data, the authors highlighted that, although the vagus nerve increased the performance of offline spatial working memory tasks, there was no evidence of improvement of vagal stimulation on digit working memory tasks.

In summary, our Research Topic provides very interesting studies that add reliable data to better understand the role of the vagus nerve in health and disease in experimental and clinical conditions.

## Author contributions

VV draft the editorial and gave final approval.

## Conflict of interest

The author declares that the research was conducted in the absence of any commercial or financial relationships that could be construed as a potential conflict of interest.

## Publisher's note

All claims expressed in this article are solely those of the authors and do not necessarily represent those of their affiliated organizations, or those of the publisher, the editors and the reviewers. Any product that may be evaluated in this article, or claim that may be made by its manufacturer, is not guaranteed or endorsed by the publisher.
